# Supplementary material for: Re-testing as a method of implementing external quality assessment program for COVID-19 real time PCR testing in Uganda
Source: PLoS One. 2024 Jan 24;19(1):e0287272. doi: 10.1371/journal.pone.0287272 (PMC10807774; doi:10.1371/journal.pone.0287272)
Supplement: Attachment — Submitted filename: PLOSONE REVIEW.docx [file pone.0287272.s002.docx]

**REVIEW: RE-TESTING AS A METHOD OF IMPLEMENTING EXTERNAL QUALITY ASSESSMENT PROGRAMME FOR COVID-19 PCR TESTING IN UGANDA**

1. What type of PCR? Is it conventional or real time PCR? This should be stated at required points in the paper.
2. There is need to check the use of the word “quadrille” and use as appropriate.
3. The statement starting from “In September …” needs to be referenced.
4. The paragraph starting from "A total of eight different genes, dyes …” is ambiguous and disjointed. It is important to state clearly the functons of the primers and the dyes. The dyes are not the targets.
5. Abbreviations are not expected at the beginning of a sentence as seen in the statement starting from “CT …”
6. In what form were the samples retrieved? As extracted DNA or in the crude form? What was the storage condition at the different facilities prior to retrieving the samples from them? How many days after initial testing were the samples re-tested?
7. Quotation referred to J. Greenman *et. al.* needs to be revised for clarity.
