# Supplementary material for: Re-testing as a method of implementing external quality assessment program for COVID-19 real time PCR testing in Uganda
Source: PLoS One. 2024 Jan 24;19(1):e0287272. doi: 10.1371/journal.pone.0287272 (PMC10807774; doi:10.1371/journal.pone.0287272)
Supplement: Attachment — Submitted filename: Response to Reviewers(1).docx [file pone.0287272.s004.docx]

Response to Reviewers

Reviewer #1: 1. Previous corrections such as the use of abbreviations at the beginning of a sentence is still in the manuscript.

Response: Thank you for the guidance; abbreviations locally used such as Ministry of Health (MoH) and Uganda Virus Research Institute (UVRI) have been written in full in the abstract. However, there are universally accepted abbreviations such as COVID19, PCR that the target audience for this manuscript are conversant with.
2. The word quadrilled was still used wrongly in the manuscript.

Response: Thank you for this observation. However, the word “quadrilled” does not exist in this revised manuscript to the best of my knowledge. Deliberate efforts have been put to avoid colloquial words in the revised manuscript.

3. CT value means cyclethreshold and should be referred to as such. Thermocycler value cannot be abbreviated as CT.

Response: Thank you so much for this correction. What was previously written as Thermocycler value has been changed to cycle Threshold in the entire revised manuscript as guided

4. The use and interpretation of dyes and genes is still confusing in this manuscript. For clarity, dyes are the colored probes used to detect the genes intended for identification (amplification). Dyes are not to be grouped as items to be identified as this paper implied especially summarized in the pie chart.

We take note of your comment. The entire section of genes and dyes has been re-written in the revised manuscript. I believe the ambiguity has been clarified in the revised manuscript.

Reviewer #2: While the manuscript has improved, there are important comments from myself and other reviewers that have not been adequately addressed. I have some examples below, but I recommend that the authors carefully go through the prior detailed review to make sure they understand and address each comment. The manuscript contains valuable data, but there are still important inaccuracies in the analysis and interpretations. Just a couple of examples:

-A fundamental problem with the paper is the expectation that the specific Ct values produced by the reference lab should match the specific Ct values produced by each other lab. This is not true, because machines and assays have different ways of producing and measuring fluorescence, which are not expected to generate the same Ct value for the same sample. Here is an example of a study that demonstrates variable Ct values across assays and platforms, even though all are valid to use:
https://journals.asm.org/doi/10.1128/jcm.00821-20
Because of this well-known inter-assay variability, specific comparisons are not appropriate, though the relative value of each sample can be compared e.g. using correlation tests.

Response: Thank you for this observation and comment. In the dissuasion of the revised manuscript, we acknowledge inter-assay differences as a possible cause of the discrepancies. Much as the exact values cannot be reproduced across different platform, there should be an acceptable range of values which are usually +/- 2SD. However, results from the UVRI are treated as the true results by the National Quality assurance committee after weighing in so many variables

-I still do not understand Figure 1. Using the external site MNRH as an example (first set of bars), it looks like 7 samples tested positive at MNRH and 10 samples tested positive at UVRI. This would mean that there were either 3 false-positives at UVRI or 3 false-negatives at MNRH. This is the opposite of what is described in the text.

Response: Thank you for this keen observation. The confusing graph has been removed and new one plotted as reflected in the revised manuscript. The values were assigned a wrong tittle while inputting.

-As reviewers 1 and 2 noted, the description of dyes and probes in the various real-time PCR assays is confusing and misleading. Dyes are not probes; each assay will use a dye, and some will also use a probe. By placing them in the same pie chart in Figure 2, the authors make it seem as though each assay uses either a dye or a probe.

Response: This section has been extensively revised, we believe your issue has been addressed in the revised manuscript.

-Again, these are just a few examples of where I do not think the authors have adequately addressed prior comments.

Response: We believe the revised manuscript addresses most of your concerns and questions. I would be much excited if the scientific reviewers can clear this paper for publication before it is taken by events.
